# Maternal peripheral blood level of IL-10 as a marker for inflammatory placental malaria

**DOI:** 10.1186/1475-2875-7-26

**Published:** 2008-01-29

**Authors:** Edward R Kabyemela, Atis Muehlenbachs, Michal Fried, Jonathan D Kurtis, Theonest K Mutabingwa, Patrick E Duffy

**Affiliations:** 1 Mother Offspring Malaria Studies (MOMS) Project, Seattle Biomedical Research Institute, Seattle WA 98109, and Muheza Designated District Hospital, Muheza, Tanzania; 2 Tumaini University, Moshi, Tanzania; 3 University of Washington, Seattle WA 98195, USA; 4 Brown University, Providence, RI, USA; 5 National Institute for Medical Research, Dar es salaam, Tanzania

## Abstract

**Background:**

Placental malaria (PM) is an important cause of maternal and foetal mortality in tropical areas, and severe sequelae and mortality are related to inflammation in the placenta. Diagnosis is difficult because PM is often asymptomatic, peripheral blood smear examination detects parasitemia as few as half of PM cases, and no peripheral markers have been validated for placental inflammation.

**Methods:**

In a cohort of Tanzanian parturients, PM was determined by placental blood smears and placental inflammation was assessed by histology and TNF mRNA levels. Maternal peripheral blood levels of several immune mediators previously implicated in PM pathogenesis, as well as ferritin and leptin were measured. The relationship between the levels of these soluble factors to PM and placental inflammation was examined.

**Results:**

Peripheral levels of TNF, TNF-RI, TNF-RII, IL-1, IL-10, and ferritin were elevated during PM, whereas levels of IFN-γ, IL-4, IL-5 and IL-6 were unchanged and levels of leptin were decreased. In receiver operating characteristic curve analysis, IL-10 had the greatest area under the curve, and would provide a sensitivity of 60% with a false positive rate of 10%. At a cut off level of 15 pg/mL, IL-10 would detect PM with a sensitivity of 79.5% and a specificity of 84.3%. IL-10 levels correlated with placental inflammatory cells and placental TNF mRNA levels in first time mothers.

**Conclusion:**

These data suggest that IL-10 may have utility as a biomarker for inflammatory PM in research studies, but that additional biomarkers may be required to improve clinical diagnosis and management of malaria during pregnancy.

## Background

Placental malaria (PM) due to *Plasmodium falciparum *is a major cause of mortality for mothers and their offspring, and is most frequent and severe during first pregnancies [1]. PM is caused by parasite-infected erythrocytes that bind to chondroitin sulfate A (CSA) and sequester in the placenta [2]. In histologic studies, PM can appear as an acute condition with little to no inflammation, or as a chronic disorder with sometimes heavy inflammation and deposition of parasite haemozoin (also called pigment) [3]. Chronic inflammatory PM has been most closely related to poor maternal and foetal outcomes in earlier studies [4]. In areas of stable malaria transmission, first time mothers often develop chronic PM, with inflammatory infiltrates and elevated Type 1 cytokines in the placenta [4,5].

Antenatal diagnosis of PM by Giemsa-stained blood smears fails to identify a substantial proportion of PM cases [6], possibly as many as half [1] and no tools exist that can predict poor pregnancy outcomes. PCR-based detection of *P. falciparum *DNA in peripheral blood is frequently positive when peripheral blood smear is negative. However, PCR can detect dead parasites, free parasite DNA, or DNA in phagocytic cells, and PCR-detection is not associated with pregnancy outcomes [6]. Antigen capture tests show promise, but they yield information only on parasitaemia and not inflammation [7]. A recent study from Kenya reported an association between plasma urokinase receptor levels measured at delivery and low birth weight in maternal malaria [8], suggesting that host biomarkers may be useful for discriminating women likely to experience poor outcomes from other women. Peripheral biomarkers of placental inflammation may be of particular value, since this condition is related to poor outcomes. In the present study peripheral blood levels of several immune mediators and other proteins in a cohort of Tanzanian women was examined at the time of delivery, and their associations with PM and placental inflammation was determined.

## Methods

### Clinical procedures

Placental samples, peripheral blood and clinical information were provided by Tanzanian women aged 18 to 45 years delivering at the Muheza Designated District Hospital, Muheza, Tanga region, in an area of intense malaria transmission. These women were participating in a birth cohort study known locally as the Mother-Offspring Malaria Studies (MOMS) Project. Women signed an informed consent form before joining the study, and women with known HIV or HIV-related sequelae in their offspring were excluded. Routine microbiological testing for other infectious diseases was not performed at the study site. Clinical information was collected by project nurses and assistant medical officers on standardized forms. Study procedures involving human subjects were approved by the International Clinical Studies Review Committee of the Division of Microbiology and Infectious Diseases at the US National Institutes of Health, and ethical clearance was obtained from the Institutional Review Boards of Seattle Biomedical Research Institute and the National Institute for Medical Research in Tanzania.

Peripheral blood was collected in citrate phosphate dextrose around the time of delivery, and plasma was separated and frozen at -80°C. The placenta was collected at delivery, and a full thickness biopsy from the middle third of the placental disc was taken. Tissue was fresh frozen in liquid nitrogen and stored at -80°C. Placental blood samples were obtained by manual compression of the placental tissue in a grinder. Placental parasitaemia was defined as the identification of any parasites in a placental blood slide by microscopy. Thick and thin smears were prepared; thin smears were fixed with methanol. Blood slides were stained for 10 minutes in 10% Giemsa, washed in tap water, air-dried, then examined using light microscopy at 1000 × magnification. Ten thousand red cells were examined in the thin smear before concluding that a placental blood slide was negative.

### Laboratory procedures

Plasma levels of cytokines, cytokine receptors, ferritin and leptin were analyzed using a multiplexed, bead-based platform (BioPlex^®^, BioRad, Irvine, CA) and custom-made assay kits as previously described [9]. Detection limits for these assays were as follows: TNF 0.10 pg/ml, TNF receptor (R) I 1.58 pg/ml, TNF-RII 0.21 pg/ml, IFN-γ 0.04 pg/ml, IL-1 0.01 pg/ml, IL-4 0.30 pg/ml, IL-5 0.02 pg/ml, IL-6 0.45 pg/ml, IL-10 0.02 pg/ml, ferritin 0.07 ng/ml, and leptin 1.28 pg/ml. Levels of soluble factors were adjusted to account for dilution in anticoagulant at the time of sample collection. For each plasma sample, all analytes were assayed in a single day, thus eliminating freeze/thaw cycles.

For histologic analysis, PM-positive tissue was selected and 5 mm cryosections of placental tissue were fixed in methanol and stained with Giemsa. Sections were assessed by examining greater than ninety 600 × fields per section. Immune infiltrates within the intervillous spaces were qualitatively scored as (-) for none or very few inflammatory cells present, (+) for inflammatory cells present. Histological analysis was performed by a single observer (A.M.).

Quantitative PCR was performed as described elsewhere [10]. Briefly total RNA was extracted from frozen cryosections using RNeasy minikits (Qiagen) and cDNA was synthesized using Superscript III enzyme (Invitrogen) and anchored oligodT20 primers. Real-time PCR was performed in duplicate using SYBR green master mix and an ABI Prism 7000 or 7500 (Applied Biosystems). Threshold cycles (CT) were calculated and normalized to CT of KRT7 (a gene expressed by trophoblasts and not by inflammatory cells). Data are presented as fold-difference from control gene, calculated by 2^(control CT-gene CT)^. The oligonucleotide primers used for PCR reactions included: TNF Forward CACGCTCTTCTGCCTGCT; TNF-α Reverse CAGCTTGAGGGTTTGCTACA; KRT7 forward: GGCTGAGATCGACAACATCA; KRT7 reverse: CTTGGCACGAGCATCCTT.

### Statistical analysis

Student's t-test was used for the analysis of maternal age and birth weight within primigravid (first pregnancy) and multigravid (second and later pregnancy) groups. Mann-Whitney test was used to examine cytokine levels. Linear regression coefficients were calculated using simple regression analysis. Receiver operating characteristic (ROC) curve and area under the curve (AUC) analyses were performed with IL-10 and other soluble factors levels as continuous variables using JROCFIT and JLABROC4 algorithms that are available online at the URL [11]. Sensitivities and specificities of elevated IL-10 to detect PM were calculated at specific cutoff levels of 10 pg/ml, 15 pg/ml or 35 pg/ml. Other analyses were performed using Statview 5.0.1 (SAS Institute, Cary, North Carolina, United States).

## Results

Peripheral plasma samples used for these studies were provided by 660 women delivering singleton live-born babies in Muheza, Tanzania. Clinical data are shown in Table 1. PM+ multigravid women were younger than PM- multigravid women, and birthweight was significantly lower in PM+ deliveries compared to PM- deliveries in both gravidity groups.

**Table 1:** Characteristics of the study population. *

	**Primigravidae**			**Multigravidae**		
**Characteristic**	**PM- (n = 166)**	**PM+ (n = 39)**	**P**	**PM- (n = 415)**	**PM+ (n = 40)**	**P**

Maternal age in years (Mean; SD)	20.6 (3.3)	19.7 (1.9)	0.0964	28.9 (5.9)	25.7 (4.4)	**0.0008**
Birth weight in kg (Mean; SD)	3.10 (0.43)	2.83 (0.42)	**0.001**	3.25 (0.386)	3.04 (0.36)	**0.0014**

*Data presented are Mean (SD).

### Peripheral levels of cytokines, leptin and ferritin vary during PM

Comparison of concentrations of cytokines and other soluble factors in maternal peripheral blood stratified for PM and parity is shown in Table 2. PM significantly increased peripheral levels of TNF, TNF-RII, IL-10 and ferritin in women of both parities. Peripheral levels of TNF-RI and IL-1 significantly increased while levels of leptin significantly decreased in primigravid but not multigravid women during PM. The levels of other soluble factors were similar between PM- and PM+ women.

**Table 2:** Peripheral levels of cytokines and other soluble factors stratified by parity and PM status.*

	**Primigravidae**			**Multigravidae**		
**Factor**	**PM- (n = 166)**	**PM+ (n = 39)**	**P**	**PM- (n = 415)**	**PM+ (n = 40)**	**P**

TNF	22.9 [8.3–46.7]	62.1 [26.3–127.5]	<0.0001	18.7 [8.70–37.9]	57.7 [23.8–84.7]	0.0002
TNF- RI	948 [550–1411]	1374 [851–2290]	0.0003	812 [481–1249]	1004 [517–1580]	0.0978
TNF- RII	186 [0–494]	673 [260–1425]	<0.0001	190 [0–403]	590 [129–959]	<0.0001
IL-1	2.28 [0.72–4.36]	5.05 [1.70–11.3]	0.0018	2.06 [0.73–4.46]	3.06 [0.32–7.36]	0.1409
IL-4	0.0 [0.0-0.0]	0.0 [0.0-0.0]	0.7676	0.0 [0.0-0.0]	0.0 [0.0-0.0]	0.9054
IL-5	1.96 [0.39–4.21]	1.76 [0.12–3.32]	0.4215	2.09 [0.39–4.09]	1.90 [0.89–5.89]	0.3158
IL-6	18.4 [9.26–37.6]	29.3 [15.0–42.5]	0.083	13.1 [3.75–29.5]	13.2 [8.83–19.2]	0.7618
IL-10	5.69 [3.28–11.3]	23.4 [15.1–62.7]	<0.0001	6.12 [2.91–11.6]	22.2 [13.9–40.3]	<0.0001
IFN-γ	0.0 [0.0-0.0]	0.0 [0.0–3.67]	0.5083	0.0 [0.0-0.0]	0.0 [0.0-0.0]	0.6607
Leptin	2404 [973–6218]	1029 [658–4115]	0.0245	2161 [1014–5293]	1490 [452–5052]	0.3444
Ferritin	14.0 [8.00–32.4]	62.7 [20.8–144.8]	<0.0001	11.6 [6.7–25.9]	40.4 [19.3–82.0]	<0.0001

*Data are presented as median [interquartile ranges]. P-values were calculated by Mann-Whitney test.

### Peripheral IL-10 levels are markers of PM and placental inflammation

**Figure 1:**
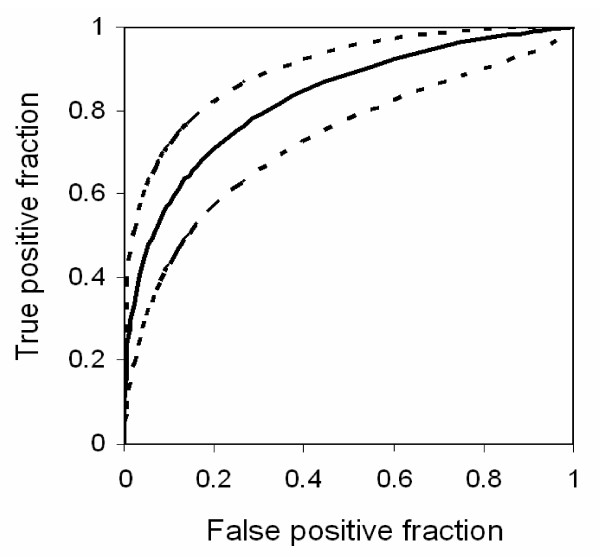
Receiver operator curve for peripheral IL-10 levels in first time mothers to detect PM. Solid line is the best fit curve; dashed lines show the 95% confidence intervals.

The soluble factors that were significantly elevated in peripheral blood during PM were analyzed by ROC curve analysis to determine their utility as biomarkers to detect PM (Table 3). IL-10 had the greatest area under the curve (AUC) at 0.83 in first time mothers and 0.82 for all mothers, indicating the highest sensitivity and specificity. The ROC curve for IL-10 in first time mothers is shown in Figure 1. Using an IL-10 cutoff for a false positive rate of 10% would yield a sensitivity of 60%, whereas a cut off for sensitivity of 90% would yield a false positive rate of 50%. Ferritin and TNF-RII had AUC values greater than 0.75 in first time mothers. Derived values, resulting from the combination by summation or addition of IL-10 with either ferritin or TNF-RII provided no improvement in sensitivity and specificity (data not shown).

**Table 3:** Area under the Receiver Operator Characteristic (ROC) curve to detect PM.*

**Soluble factor**	**All gravidities**	**Primigravidae**
TNF	0.690	0.731
TNF- RI	0.635	0.694
TNF- RII	0.731	0.752
IL-1	0.608	0.658
IL-10	0.815	0.830
Ferritin	0.733	0.759

*Only cytokines and other soluble factors significantly elevated during PM are shown.

**Figure 2:**
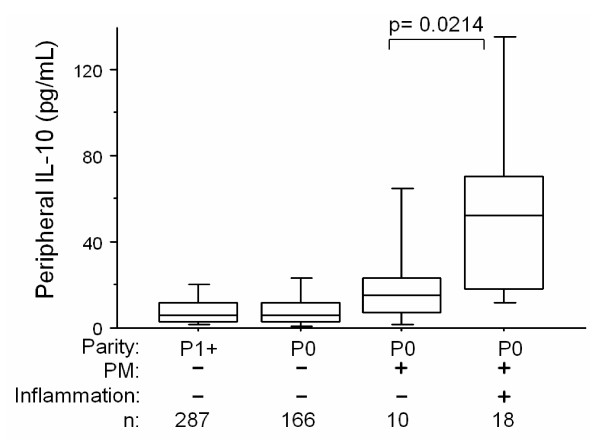
Peripheral IL-10 levels stratified for maternal parity, PM and the presence of inflammatory cells by placental histology. P-value was calculated using Mann-Whitney test. P0, primigravidae; P1+, multigravidae.

**Figure 3:**
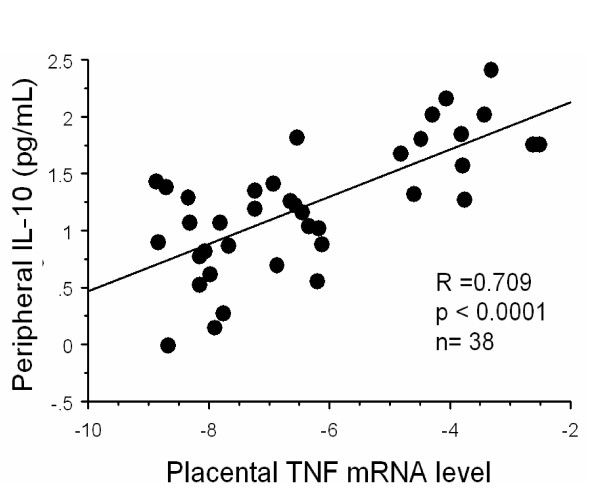
Relationship of peripheral IL-10 levels and placental TNF-α mRNA levels in first time mothers. Gene expression is presented as 2^x ^fold expression over KRT7. Simple regression analysis was used to calculate R and P-values.

The ability of IL-10 elevations above various threshold values to discriminate infected from uninfected women was examined (Table 4). An IL-10 cutoff level of 15 pg/mL yielded values above 75% for both parameters. Peripheral IL-10 levels were specifically elevated in first time mothers who had placental inflammation by histology (Figure 2). Further, peripheral IL-10 levels correlated significantly with placental TNF mRNA (Figure 3).

**Table 4:** Sensitivity and specificity of discrete IL-10 cut-off levels toclassify cases of PM in first time mothers (n = 205).

**IL -10 levels**	**Sensitivity (%)**	**Specificity (%)**
≥ 10 pg/mL	84.6	72.9
≥ 15 pg/mL	79.5	84.3
≥ 35 pg/mL	43.6	95.8

## Discussion

Peripheral blood smear analysis has low sensitivity to detect PM. PCR based and antigen capture tests for the diagnosis of PM have increased sensitivity but cannot detect inflammation, which is related to poor pregnancy outcomes. This study suggests that peripheral IL-10 levels may be a useful tool to identify women with inflammatory PM and therefore those likely to have poor pregnancy outcomes. Using a cut-off level of 15 pg/mL, IL-10 levels would detect PM with a sensitivity of 79.5% and specificity of 84.3%. IL-10 may have utility in longitudinal studies, examining the burden of malaria over gestation, when the placenta is not available for microscopic analysis. Future studies should measure IL-10 levels throughout gestation to assess relationships to antenatal parasitemia and to pregnancy outcomes.

IL-10 is a key cytokine both in protection and immunopathology during malaria. High levels of IL-10 observed during malarial episodes may be beneficial by reducing the inflammatory response, but may be detrimental by decreasing antiparasitic cellular immune responses. IL-10 is an anti-inflammatory cytokine that acts in part by blocking monocyte/macrophage production of inflammatory cytokines such as IL-6, TNF, and IL-l [12]. Animal studies have suggested that IL-10 may play a regulatory role during parasitic infection that modulates susceptibility. In particular, IL-10 inhibits the microbicidal activity of IFN-γ-treated macrophages against intracellular parasites such as *Toxoplasma gondii *[13], *Trypanosoma cruzi *[14] and *Leishmania major *[15] and the killing of extracellular *Schistosoma mansoni *schistosomulas [16]. These effects may result from decreased production of the toxic nitrogen oxide metabolites[17].

The blood stages of *P. falciparum *are also cleared by phagocytosis and killed by oxidative products of nitric oxide released by macrophages [18]. IL-10 has been previously observed to be elevated during malarial episodes in non-pregnant [19,20] and pregnant individuals [21]. Both increased and decreased levels of IL-10 have been associated with poor malaria outcomes. Low levels of IL-10 or low IL-10 to TNF ratios were associated with severe malarial anemia in African children [22,23] while high IL -10 levels were associated with reduced ability to eliminate malaria parasitaemia in Tanzanian children [24].

PM results from the accumulation of parasites that bind to CSA in the intervillous spaces of the placenta [2,25]. In response to the sequestered mass of parasites, inflammatory cells infiltrate the intervillous spaces This inflammatory infiltrate can be massive, and prominently features monocytes/macrophages. In vitro data suggests these cells are the principal source of IL-10 [21]. In Kenyan children, high levels of peripheral blood IL-10 were positively correlated with binding of infected red blood cells to CD36 [26], but the relevance of this observation to malaria pathogenesis is unknown, and we find that levels of IL-10 also increase when CSA-binding parasites are the major parasite form causing infection. Placental levels of TNF increase during PM [5,21,27] and TNF gene expression is specifically related to placental inflammation [10]. Increased placental blood levels of TNF are related to poor outcomes for both the mother and her newborn [5,27]. In the present study, placental TNF mRNA positively correlated to peripheral blood IL-10 levels in first-time mothers, strengthening the association between peripheral IL-10 levels and placental inflammation.

The present data indicate that peripheral ferritin levels are also elevated during PM. Ferritin is a positive acute phase protein and is known to increase during infection and injury. In non-pregnant individuals, ferritin levels increase during both asymptomatic and symptomatic malaria, and the highest levels have been recorded in individuals with severe disease [28]. Serum ferritin may also increase in the presence of subclinical infection [29]. During the acute phase response, inflammatory cytokines such as IL-1β increase the synthesis of both heavy and light subunits of ferritin [30]. In this Tanzanian cohort, PM was associated with elevated levels of IL-1 and TNF in maternal peripheral blood, particularly among first time mothers who are most likely to experience placental inflammation. Ferritin is widely used for determining iron deficiency anemia in industrialized countries, and therefore has the advantage of existing diagnostic platforms. For this reason, ferritin should also be evaluated in prospective studies as a cost-effective antenatal assay for screening inflammatory PM and poor pregnancy outcomes in tropical countries.

## Conclusion

In summary, these data suggest that the peripheral IL-10 level may be useful as a biomarker of inflammation due to PM. Future studies should measure antenatal levels of IL-10, and assess its relationship to parasitemia and pregnancy outcomes, and its utility for monitoring interventional trials. The sensitivity and specificity of peripheral IL-10 levels at delivery suggest that they may not be sufficient to be used clinically as diagnostic tools. Additional biomarkers of PM, placental inflammation and PM-related poor outcomes are needed to improve the clinical management of this major public health problem.

## Authors' contributions

TKM, MF, and PED designed and managed the MOMS Project. ERK and JDK performed the multiplex cytokine assay. AM performed PCR and histology studies. AM analyzed the data and wrote the manuscript with assistance from other authors.

## Conflict of interest

The author(s) declare that they have no competing interests.
